# Rational Design of a Molecularly Imprinted Sensor on a Biomass Carbon Platform for Glyphosate Monitoring in Traditional Chinese Medicines

**DOI:** 10.3390/polym18010021

**Published:** 2025-12-22

**Authors:** Xin Wang, Delai Zhou, Xuxia Liu, Guodi Lu, Jia Hou, Jian Xu, Fude Yang

**Affiliations:** 1 College of Pharmacy, Gansu University of Traditional Chinese Medicine, Lanzhou 730101, China; wangxinnj@yeah.net (X.W.); gszyzdl2022@163.com (D.Z.); gszylxx6823@163.com (X.L.); luguodi@126.com (G.L.); houjia2012@163.com (J.H.); 2 Research Center for Natural Medicine and Chemical Metrology, Lanzhou Institute of Chemical Physics, Chinese Academy of Sciences, Lanzhou 730000, China; xujian1980@licp.cas.cn

**Keywords:** molecularly imprinted polymer, glyphosate, electrochemical sensor, biomass-derived carbon, Traditional Chinese Medicine analysis

## Abstract

A molecularly imprinted electrochemical sensor was developed for the selective and sensitive detection of glyphosate in Traditional Chinese Medicine samples. An excellent conductive hierarchical porous carbon substrate made from sodium alginate and ammonium chloride co-carbonization was used to build the sensor. The molecularly imprinted polymer layer was systematically designed using Density Functional Theory calculations, which identified nicotinamide as the optimal functional monomer. A deep eutectic solvent was utilized as an effective green eluent for template removal. Under optimized conditions, the sensor demonstrated a wide linear detection range from 1.0 × 10^−9^ to 1.0 × 10^−6^ M with an exceptionally low detection limit of 8.8 × 10^−10^ M. The sensor exhibited satisfactory reproducibility (RSD = 3.35%, *n* = 6), repeatability (RSD = 5.0% over 6 cycles), and robust stability (retaining >90% initial response after 10 days). The sensor displayed satisfactory recovery rates of 94.47–112.23% and RSD values ranging from 1.37–3.01% when applied to real traditional Chinese medicine samples, thereby confirming its accuracy and practical utility for glyphosate residue analysis in complex matrices. This study introduces an effective sensing platform that integrates rational design principles with environmentally friendly synthesis strategies for quality control in traditional medicine applications.

## 1. Introduction

Glyphosate (Gly), a non-selective post-emergence wide-spectrum herbicide, is extensively utilized to mitigate the effects of weeds and pests on agricultural production [1]. Nonetheless, Gly exposure may result in enduring harm to human health [2]. The U.S. Environmental Protection Agency has shown that Gly may increase the occurrence of kidney carcinoma in mice. The International Agency for Research on Cancer also indicated that Gly is a carcinogenic substance for humans [3,4]. Consequently, many countries are reassessing glyphosate use and allowable levels. For example, EU food MRLs range from 0.05 mg/kg for most animal products up to 20 mg/kg for certain crops (e.g., soybean, sunflower, barley, oat) [5]; China’s national food-safety standards set MRLs between 0.05 and 5 mg/kg [6]; and the U.S. EPA’s maximum limit for glyphosate in water is 700 μg/L [7].

Following the prohibition of paraquat in China in 2016, the domestic manufacture and application of glyphosate have escalated, particularly in the cultivation of traditional Chinese medicinal (TCM) plants [8]. With the increasing prominence of traditional Chinese medicine, concerns about its safety are also rising [9,10]. However, due to the complex nature of traditional Chinese medicine, pesticide residue testing, particularly for glyphosate, has become a significant challenge.

Gly is polar, resulting in its high solubility in water. Furthermore, it lacks both chromophore and fluorophore moieties. Consequently, various Gly derivatization methods were developed for chromatographic assays [11]. Liquid and gas chromatography [12], together with capillary electrophoresis [13], were integrated with mass spectrometry [14] or electrochemiluminescence detection [15] to address this issue. Nonetheless, these techniques are arduous and necessitate costly apparatus and proficient operators. Electrochemical sensors are highly promising for quantifying target analytes in practical applications due to their relatively low development expenses, user-friendliness, and potential for downsizing into portable devices [16]. However, sensors used for Gly determination may be affected by limited selectivity.

Molecular imprinting polymers (MIPs) mimic the interactions between enzymes and substrates or antibodies and antigens, enabling specific recognition of analytes or template molecules [17,18]. MIP sensors are created through the copolymerization of analyte and functional monomers on the sensor [19]. Upon the removal of the analyte, spaces that correspond to the structure and size of the analyte will form on the membrane, enabling the MIP sensor to specifically identify the analyte [20]. The distinct characteristics of structure predictability, recognition specificity, and stability have led to the widespread application of MIPs in numerous high-sensitivity sensors [21,22,23]. Therefore, MIPs-based electrochemical sensors serve as an optimal platform for applications necessitating high selectivity in complex samples [24], particularly for the accurate detection and quantification of pesticide residues within the complex compositions of TCM.

The matrix and structure of MIPs-based electrochemical sensors are critical factors influencing sensor performance [25]. An appropriate substrate must exhibit a substantial specific surface area to enhance imprint site density, superior conductivity to promote electron transport, and abundant surface chemistry to guarantee stable adhesion of the MIPs layer [26,27]. Researchers have extensively investigated various nanomaterials to meet these requirements, including highly conductive MXenes [28,29], metal–organic frameworks (MOFs) [30], reduced graphene oxide (rGO) [31], and their composites with metal nanoparticles like gold and platinum [32,33]. The materials have significantly improved sensor performance. While the above nanocomposites greatly improve the performance of sensors, their growth still faces problems related to cost, sustainability, and being beneficial for the environment. For example, the preparation of MXenes requires the use of hazardous hydrofluoric acid etching and is susceptible to oxidation [34], whereas graphene-precious metal composites are excessively costly. These factors taken together constrain their potential for usage in practice.

Based on careful considerations of sustainability and practicality, this study selected and developed porous carbon materials derived from biomass sodium alginate as sensor substrates. The carbonization preparation method utilized is less harsh and more environmentally sustainable, consistent with the evolving focus of green analytical chemistry. Carbon materials with a hierarchical porous structure were successfully constructed. This three-dimensional network provides a significant specific surface area, facilitating the molecular imprinting process, and demonstrates excellent electronic conductivity following high-temperature carbonization. Additionally, the numerous functional groups, including hydroxyl and carboxyl groups, present on the carbon surface establish strong interfacial bonds with the molecular imprinting precursor, thereby effectively inhibiting the delamination of the molecularly imprinted layer during operation.

**Scheme 1:**
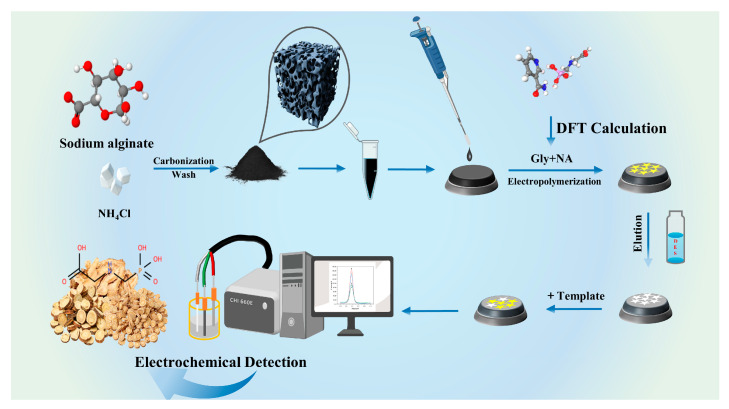
Workflow: MIP sensor fabrication and electrochemical detection of Gly.

This research details the creation of an electrochemical platform utilizing molecular imprinting strategies and biochar materials for the detection of Gly in TCM materials. The uniqueness of our strategy is not in an individual component but in the synergistic amalgamation of multiple strategically selected pieces. Single-biomass (sodium alginate) porous carbon materials were initially utilized to fabricate the working electrode, aiming to enhance its performance. Density functional theory (DFT) utilizing the Dmol^3^/GGA-PBE/DNP basis set was applied to identify a suitable monomer unit for integration with Gly molecules in the formation of an MIP film. Deep eutectic solvents (DES) have been introduced as a sustainable and efficient alternative to traditional organic eluents. The electrochemical detection of Gly utilized potassium ferricyanide (K_3_Fe(CN)_6_) as a redox signaling probe. This sensor’s efficacy is validated through its successful application in quantifying Gly in complex TCM extracts, highlighting its potential as a practical instrument for ensuring the safety and quality of these products. Scheme 1 shows the workflow for fabricating the MIP-modified electrochemical sensor and its use for detecting Gly.

## 2. Materials and Methods

### 2.1. Chemicals and Materials

Sodium alginate (SA), ammonium chloride (NH_4_Cl), potassium ferricyanide (K_3_[Fe(CN)_6_]), nicotinamide (NA), glyphosate (Gly), ethylene glycol (EG), choline chloride (ChCl), sulfuric acid (H_2_SO_4_), disodium hydrogen phosphate (Na_2_HPO_4_), sodium dihydrogen phosphate (NaH_2_PO_4_), hydrochloric acid (HCl), sodium hydroxide (NaOH), and other reagents were analytical grade and bought from Aladdin (Shanghai, China). Chinese herbal slices (Licorice root, Radix angelicae sinensis, Manyinflorescenced Sweetvetch Root) were provided by the Affiliated Hospital of Gansu University of Chinese Medicine. 0.1 M phosphate buffer solutions (PBS) with a pH of 5.0 to 9.0 were prepared by 0.1 M NaOH and 0.1 M HCl, respectively. DES was prepared by ultrasonication of ChCl and EG at a molar ratio of 1:3 for 30 min at 60 °C.

### 2.2. Apparatus

The microscopic morphologies and elemental compositions of Porous Biomass Charcoal (PBC) were determined by scanning electron microscopy (SEM; ZEISS Sigma 300, Oberkochen, Germany) coupled with energy dispersive spectroscopy (EDS; OXFORD Xplore, Abingdon, UK). Fourier-transform infrared spectroscopy (FTIR; Thermo Nicolet 380, Waltham, MA, USA), and X-ray photoelectron spectroscopy (XPS; Thermo Scientific K-Alpha, Waltham, MA, USA) were used to determine chemical bonding and elemental valence information. The pore structures were measured by N_2_ adsorption−desorption isotherms at 77 K using an automated sorption analyzer (Micromeritics ASAP 2460, Norcross, GA, USA). The structural properties of PBC were characterized by Raman spectroscopy (Horiba LabRAM HR Evolution, Kyoto, Japan). All electrochemical experiments, including cyclic voltammetry (CV), differential pulse voltammetry (DPV), and electrochemical impedance spectroscopy (EIS), were performed on a CHI660E electrochemical workstation (Austin, TX, USA) equipped with a three-electrode system. This system comprised a working electrode, a Hg/Hg_2_Cl_2_ electrode (reference electrode), and a platinum wire electrode (counter electrode).

### 2.3. Computer Simulation

In this work, Material Studio 2020 (Accelrys, San Diego, CA, USA) software was used to study the molecular imprinting systems at the atomic level. The simulation was performed using the DFT program Dmol^3^ in MS. The physical wave functions were expanded in terms of numerical basis sets, Dmol^3^/GGA-PBE/DNP(3.5) basis set. The core electrons were treated with DFT semi-core pseudo potentials. The exchange-correlation energy was calculated with Perdew-Burke-Ernzerhof (PBE) generalized gradient approximation (GGA) [35]. The COSMO continuum model, for the simulation of the dielectric constant of the solvent water, was used in the synthesis environment [36]. Geometry optimizations and energy calculations were converged using the following thresholds: SCF tolerance 1.0 × 10^−6^ Ha per atom, energy tolerance 1.0 × 10^−5^ Ha per atom, maximum force 0.002 Ha/Å, and maximum displacement 0.005 Å.

### 2.4. Synthesis of PBC

In accordance with the methodology outlined in the literature [37], SA and NH_4_Cl were incorporated into 50 mL of deionized water at a mass ratio of 1:7. Following ultrasonic treatment to achieve a uniform transparent solution, vigorous stirring was conducted at room temperature for a duration of 2 h. The solution was concentrated with a rotary evaporator and subsequently dried at 60 °C for 12 h to yield the solid. The solid was pulverized and subjected to carbonization at 900 °C for 3 h under a nitrogen atmosphere, with a heating rate of 5 °C·min^−1^. The powder was immersed in a 0.5 M H_2_SO_4_ aqueous solution at 80 °C overnight, followed by filtration, rinsing with distilled water, and drying at 60 °C to obtain the final PBC black powder.

### 2.5. Construction of PBC/GCE

The bare GCE (diameter: 3 mm) underwent pretreatment as outlined in the literature [38], which involved mechanical polishing, ultrasonic cleaning, and electrochemical activation. 1 mg of newly synthesized PBC was dispersed in 1 mL of deionized water, followed by the addition of 10 μL of Nafion solution to the nanomaterial dispersion. To ensure overall uniformity of the nanomaterials, the electrode material underwent ultrasonic treatment. An 8 μL droplet of the prepared electrode slurry was deposited onto the surface of the GCE. The modified GCE was subsequently dried at room temperature, which improved the adhesion strength of the electrode material to the GCE surface due to the incorporation of the binder (Nafion) [39].

### 2.6. Construction of MIP/PBC/GCE

Using CV, a molecularly imprinted polymer was formed on the surface of a PBC-modified GCE by electropolymerization in a 0.1 M PBS (pH 6.0) solution containing 4.0 mM Gly and 6.0 mM NA. The polymerization was conducted with 20 cycles at a scan rate of 100 mV/s over a potential range of −0.2 V to 0.8 V. The GCE was finally eluted with DES for 10 min to obtain the MIP/PBC/GCE. As a control, a NIP/PBC/GCE was prepared under identical experimental conditions, except without Gly addition.

### 2.7. Electrochemical Measurements

Prior to the electrochemical measurement, the modified electrodes were incubated in PBS with a specific concentration of Gly for 10 min, subsequently rinsed with ultrapure water. All electrochemical experiments were conducted in a 0.1 M KCl solution containing 5.0 mM of K_3_Fe(CN)_6_. The CV was performed throughout a potential range of −0.2 V to 0.8 V at a scan rate of 100 mV/s, while the EIS was executed within a frequency range of 100 kHz to 1 Hz, commencing at an initial potential of 0.198 V. The DPV responses were documented from −0.2 V to 0.8 V, with a potential increment of 4 mV, a pulse width of 0.05 s, a pulse amplitude of 0.05 V, and a pulse period of 0.5 s [40].

### 2.8. Preparation of Real Samples

**Scheme 2:**
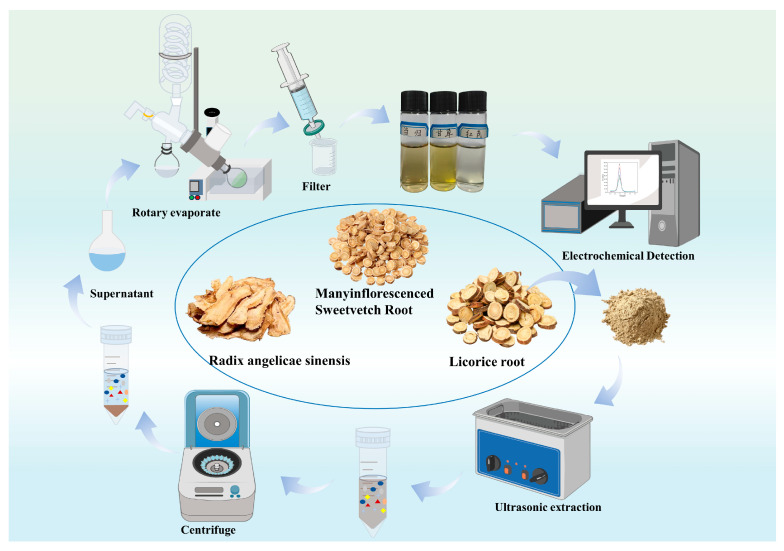
Diagram of the Real Workflow for Sample Processing.

Grind the Chinese herbal components and strain through a 100-mesh filter. Dissolve 1 g of powder in 20 mL of a 7:3 ethanol-water solution. Sonicate at 60 °C for 30 min, shaking every 5 min to ensure uniform dispersion of the solution. Centrifuge the extract at 6000 rpm for 20 min. Concentrate the supernatant to 1–2 mL using a rotary evaporator. Add PBS (pH 7.0) to a final volume of 10 mL. Filter through a 0.22-micron membrane filter and store at 4 °C for subsequent use. Scheme 2 shows the real workflow for sample processing.

## 3. Results and Discussion

### 3.1. Materials Characterization

**Figure 1:**
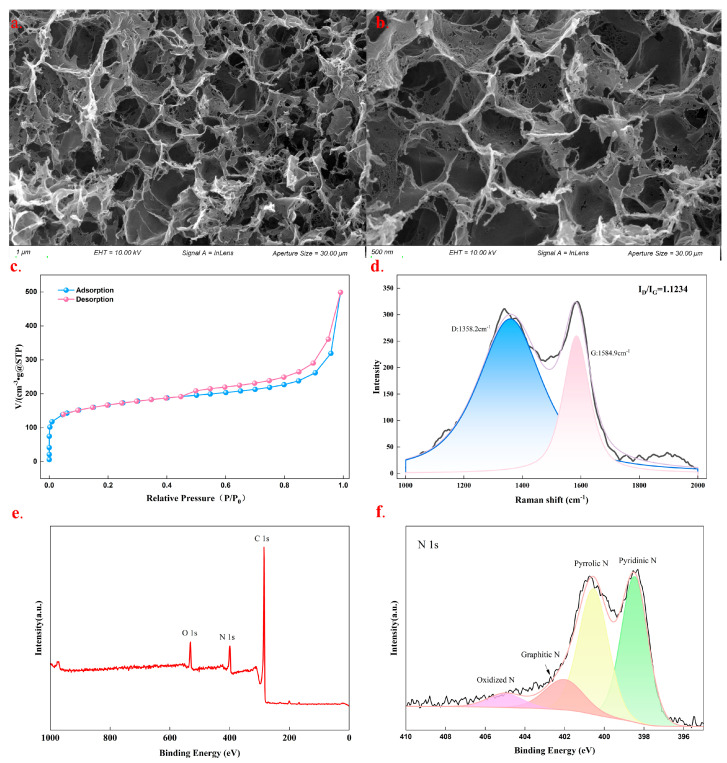
(**a**,**b**) are SEM images of PBC; (**c**) N_2_ adsorption and desorption isotherms; (**d**) Raman spectra; (**e**) XPS survey spectra; (**f**) High-resolution N 1s XPS spectra.

PBC was synthesized from SA and NH_4_Cl through an in situ bubble foaming method. With rising temperature, NH_4_Cl decomposed into NH_3_ and HCl, both of which functioned as internal pore generators. The gases formed in situ experienced volume expansion and subsequently escaped, resulting in the development of a highly interconnected macropore network structure with a specific surface area of 598.35 m^2^/g. The SEM results validated the experimental strategy (Figure 1a,b). N_2_ adsorption and desorption measurements were performed to characterize the porous structures and surface area. The N_2_ adsorption–desorption isotherm of PBC, illustrated in Figure 1c, exhibited a type IV profile characterized by an H3 hysteresis loop, indicating the mesoporous nature of PBC. The presence of a macroporous structure was confirmed by the significant increase in nitrogen adsorption volume at elevated relative pressure. The pore size distribution curve (Figure S4) indicated that PBC exhibited a characteristic mesoporous structure. The SEM observation and BET measurement provided evidence for the presence of macropores and mesopores. Macroporous structures demonstrate swift molecular adsorption and diffusion, whereas mesoporous structures offer a high specific surface area. The properties of PBC provide a solid foundation for the development of molecularly imprinted polymers.

XPS survey results verified the presence of carbon, nitrogen, and oxygen, with the detection of C–N bonds indicating successful nitrogen integration into the carbon material. The prominent C–C/C=C peaks indicate that the material has a sp^2^-dominated carbon backbone, facilitating rapid electron transport. The notable presence of C–O/C–N and C=O peaks suggests that the material surface is abundant in oxygen- and nitrogen-containing functional groups, including hydroxyl, ether, carbonyl, and carboxyl groups, which aligns with the FT-IR results (Figure S3). High-resolution N 1s spectra of PBC identify four distinct nitrogen species: pyridine nitrogen, pyrrole nitrogen, graphitic nitrogen, and oxidized nitrogen. Graphitic nitrogen improves electron mobility in the material, whereas pyridine and pyrrole nitrogen collectively offer multiple pre-assembled anchor sites for the molecular imprinting process, resulting in the formation of additional high-affinity recognition cavities.

The Raman spectra shown in Figure 1d display notable peaks at around 1358.2 cm^−1^ and 1584.9 cm^−1^, corresponding to the D band and G band, respectively. The D band is associated with disordered carbon atoms, while the G band is related to sp^2^ hybridized graphite carbon atoms. The sample displayed low ID/IG values, signifying an increased level of graphitization and conductivity, thereby improving the electrochemical performance.

### 3.2. Selection of an Appropriate Polymer

**Figure 2:**
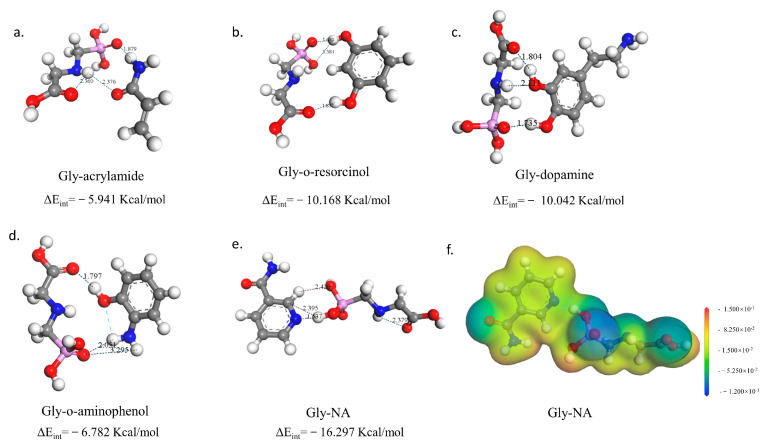
DFT theoretical calculations for interactions between monomeric units of polymers and Gly (According to stated binding energies in kcal/mol, a lower value indicates a higher binding strength), f represents the electrostatic potential map of NA-GLY.

**Figure 3:**
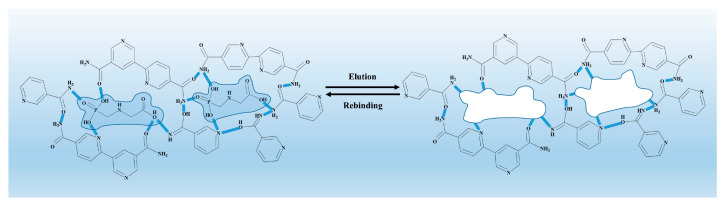
Proposed mechanism for the preparation of the MIPs and their selective recognition of Gly.

We conducted DFT calculations on the Gly unit utilizing the Dmol^3^/GGA-PBE/DNP basis set within the MS software to explore the development of more appropriate molecularly imprinted polymers. Figure 2 illustrates the optimized structural interactions between Gly and various monomer units, including nicotinamide, dopamine, acrylamide, resorcinol, and o-aminophenol. The figure presents the computed ΔE_int_ values (ΔE_int_ = E_A−B_ − (E_A_ + E_B_)), indicating the predominance of noncovalent interactions. Hydrogen bond lengths are measured in angstroms (Å), with the minimum ΔE_int_ value signifying robust interactions between the functional unit and monomer units. The Gly-NA complex, among the five monomers, demonstrates the lowest binding energy of −16.297 kcal/mol, characterized by three hydrogen bonds with lengths of 1.537 Å, 2.395 Å, and 2.420Å. We hypothesize that the relatively low binding energy of the Gly–NA complex may arise from three factors: (1) a network of three potentially cooperative hydrogen bonds, including an short contact (~1.54 Å), which could impart strong, partially stabilization; (2) geometric complementarity between nicotinamide’s CONH_2_/pyridine motifs and glyphosate’s phosphonate groups, spatial matching reduces strain and maximizes favorable contacts; and (3) electrostatic complementarity, where aligned regions of opposite potential may produce additional Coulombic attraction and induced polarization. Figure 3 illustrates the proposed MIPs integration and recognition process. We hypothesize that recognition in poly-nicotinamide MIPs arises from two cooperative features: (1) a covalently crosslinked polymer network formed during electropolymerization of pyridine (nicotinamide) units that locks cavity geometry, and (2) a complementary microenvironment within those cavities that provides multivalent, cooperative noncovalent interactions (hydrogen bonds, electrostatic attraction, π–π contacts, and local polarization). Oxidative activation of pyridine during electropolymerization generates radicals that couple and fix the spatial arrangement of CONH_2_ groups around the template; after elution, the cavities retain this 3D arrangement so that rebinding of glyphosate is driven by a network of H-bonds and electrostatic complementarity to the template’s protonation state. Collectively, these simulation results indicate that the designed electrochemical sensor delivers stable, superior molecular recognition under the tested conditions.

### 3.3. Electrochemical Characterization

**Figure 4:**
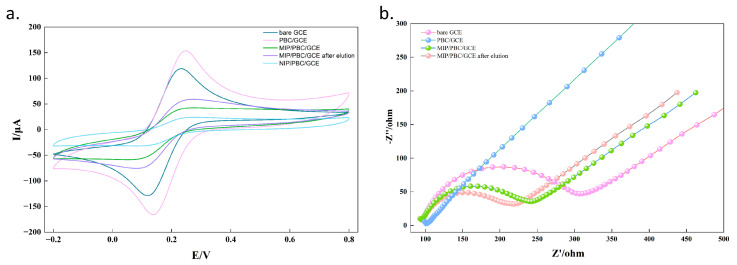
(**a**) CVs curves for stepwise modifications including bare GCE, PBC/GCE, MIP/PBC/GCE, MIP/PBC/GCE after elution, and NIP/PBC/GCE. (**b**) EIS curves for bare GCE, PBC/GCE, MIP/PBC/GCE, and MIP/PBC/GCE after elution.

This research utilized CV and EIS to analyze the electrochemical behavior of various electrodes in a redox probe solution consisting of 5.0 mM [Fe(CN)_6_]^3−/4−^ and 0.1 M KCl. The bare GCE displays symmetric redox peaks, as illustrated in Figure 4a. After PBC modification, a significant increase in peak current is observed. This increase can be linked to the enhanced active electrode area and conductivity that PBC offers, which can be attributed to the improved active electrode area and conductivity provided by PBC. Following the modification of the PBC/GCE with MIP through electrochemical polymerization, the resulting non-conductivity impeded the electron transfer of [Fe(CN)_6_]^3−/4−^, leading to a diminished peak current value. Following the elution of target molecules from the MIP, the current response surpassed that observed prior to elution. The removal of template molecules eliminated the electron-blocking layer, leaving only the molecularly imprinted cavity to facilitate electron transport. The electrochemical reaction on NIP/PBC/GCE demonstrated the lowest current response, likely attributable to the inadequate conductivity of the MIP layer, which lacks specific cavities.

The Nyquist plot (Figure 4b) reveals that the semicircular section in the high-frequency range is associated with electron transfer-limited processes, whereas the linear segment in the low-frequency range signifies diffusion-controlled processes [41]. The unmodified GCE electrode displayed the largest semicircle diameter, reflecting elevated electron transfer resistance. Following the modification of PBC on GCE, the semicircle was eliminated, leaving only a linear feature observable. After MIP electropolymerization, the semicircle diameter of the MIP-modified electrode increased significantly, thereby confirming the successful preparation of the MIP coating and leading to a reduction in response currents. Post-elution, the diameter of the electrode semicircle diminished, indicating that the MIP cavities enhanced electron conduction and lowered resistance. The slope variations in the low-frequency region of the Nyquist plot demonstrate that the PBC-modified electrode has the highest linear slope, reflecting its superior diffusion mass transfer characteristics. In contrast, the notable reduction in slope following MIP polymerization indicates that the dense polymer membrane obstructs mass transfer. The recovery of slope post-elution indicates that the creation of imprinted cavities enhances the transport of electrons and reactants, confirming the successful establishment of recognition sites. The EIS results align with the trends identified in the CV analysis. Furthermore, the effective surface area (A_eff_) of the electrode was estimated utilizing the Randles-Sevcik equation [42].

I_p_ = (2.69 × 10^5^) n^3/2^ ν^1/2^ A_eff_D^1/2^ C_0_ (1)

where I_p_, n, v, A_eff_, D, and C_0_ denote the anodic peak current, number of electrons (n = 1), scan rate (V·s^−1^), effective surface area, diffusion coefficient of the ferricyanide probe (6.70 × 10^6^ cm^2^·s^−1^), and probe solution concentration. The calculated A_eff_ values for the bare GCE and PBC/GCE are 0.079 cm^2^ and 0.165 cm^2^, respectively. This finding demonstrates that the incorporation of the PBC-modified layer markedly improves the conductivity and electrochemically active sites of the electrode.

### 3.4. Optimization Conditions

Optimizing the conditions for MIP synthesis is essential for achieving high sensitivity and selectivity of the resulting MIP toward the target analyte. To achieve ideal performance for detection, multiple experimental settings were investigated and optimized utilizing DPV with [Fe(CN)_6_]^3−/4−^ as the electrochemical probe.

#### 3.4.1. The Effect of PBC Layer Volume

**Figure 5:**
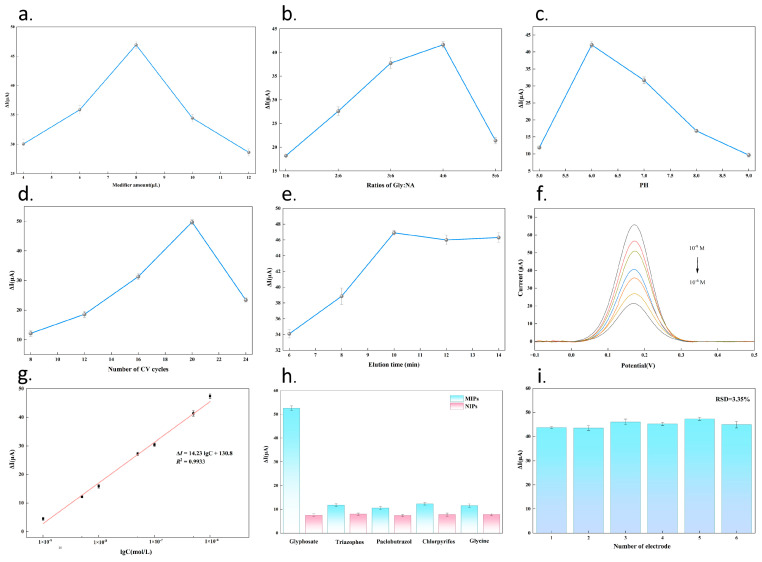
Optimization of MIP synthesis: (**a**) modifier amount, (**b**) molar ratio of Gly and NA, (**c**) pH of electrical polymerization, (**d**) number of electrical polymerization cycles and (**e**) elution time; (**f**) the corresponding linear calibration curve between the logarithm of the Gly concentration and ∆I; (**g**) DPV tests of different concentrations (10^−9^–10^−6^ M) of Gly adsorbed by MIP/PBC/GCE in 0.1 M KCl solution containing 5.0 mM [Fe(CN)_6_]^3−/4−^; (**h**) The current responses for Gly and other structural analogues; (**i**) reproducibility of the fabricated sensor.

Due to the significant link between sensing performance and coating thickness, several drop volumes were applied to the GCE surface to achieve the requisite thickness of the PBC layer, as illustrated in Figure 5a. The analysis of the difference in peak currents led to the determination of the optimal dropping volumes of 8.0 μL.

#### 3.4.2. The Effect of the Monomer to Template Ratio

The number of imprinting sites produced during the imprinting process is influenced by the ratio of functional monomers to template molecules. A reduction in the quantity of template molecules will lead to a proportional decrease in the size of the imprinting cavity. If the number of template molecules is excessive, it indicates a relatively small quantity of functional monomers, which prevents their combination with all template molecules. This also leads to a reduction in recognition sites on the imprinted film. The data indicate that optimal performance occurs at a functional monomer to template molecule ratio of 3:2 (Figure 5b), which is therefore the chosen ratio.

#### 3.4.3. The Effect of PH

The effect of pH was also investigated (Figure 5c). The sensor response is maximized at pH 6.0, demonstrating optimal molecular imprinting performance under these conditions. This arises from the robust synergistic interaction between electrostatic attraction and hydrogen bonding between Gly and NA at pH 6, resulting in the formation of the most stable complex. Deviation of pH from 6.0 alters the ionization states of both substances, thereby diminishing the critical interactions.

#### 3.4.4. The Effect of the MIP Layer Thickness

The thickness of the imprinted film is directly linked to its sensitivity and stability, which can be adjusted by controlling the number of cycles during the electrochemical polymerization process. As shown in Figure 5d, the cycle numbers of CV were optimized. The results demonstrated that the difference in peak current after elution and reattachment of the template molecules (∆I) significantly increased with the number of electrical polymerization cycles, reaching a maximum before declining after 20 cycles. This can be attributed to the insufficient number of imprinting sites in a film that is excessively thin, leading to a higher susceptibility to cracking. Undesired membrane thickness complicates the removal of template molecules anchored in the polymer, leading to low site availability and reduced capacity for binding. Consequently, 20 cycles were determined to be the optimal number for electropolymerization.

#### 3.4.5. The Effect of the Elution Time

The elution time affects the ultimate sensitivity achieved through the imprinted procedure. Insufficient elution time may result in incomplete elution, leaving unwashed template molecules. This can lead to a decrease in holes and a corresponding reduction in sensitivity. If the elution time is excessively prolonged, it may compromise the integrity of the imprinting film, resulting in decreased sensitivity. Figure 5e indicates that sensitivity peaks at an elution time of 10 min; therefore, the final elution time condition is set to 10 min.

### 3.5. Quantitative Detection of Gly

Upon elution of the constructed MIP sensor, cavities will be formed within the MIP layer that correspond to the template molecule Gly in terms of dimensions and characteristics. The recombination of the template molecule with holes impedes electron transfer, thereby diminishing the response current. Consequently, Gly can be quantitatively measured based on this principle. The DPV method was employed for the quantitative detection of Gly. Figure 5f illustrates that the response current decreased progressively with increasing Gly concentration, thereby confirming the validity of the principle. The concentration of Gly exhibited a strong linear relationship (Figure 5g) with the variation in current ΔI (ΔI = Iwashed − Iadsorbed) before and after the adsorption of Gly: ΔI = 14.23 lgC + 130.8, R^2^ = 0.9933. Additionally, it possesses an extensive detection range (1 × 10^−9^–1 × 10^−6^ M), and the limit of detection (LOD) is 8.8 × 10^−10^ M (S/N = 3). In conclusion, the sensor developed in this study demonstrates equivalent detection performance and distinct advantages compared to other electrochemical methods for Gly, as outlined in Table 1.

### 3.6. Selectivity of the Sensor

The sensor’s response was evaluated in the presence of commonly co-occurring pesticides, such as chlorpyrifos, triazophos, and paclobutrazol, as well as the structural fragment glycine, which may act as a potential interferent in TCM analysis. Figure 5h illustrates that, at equivalent detection concentrations, the sensor demonstrates a markedly enhanced response to Gly compared to the four analogues. This is attributed to the formation of specific recognition sites within the MIP films, which are tailored to complement Gly in size and shape following its removal. The structural configurations of the other four analogues do not align with these recognition sites. The control experiment demonstrated that the NIP films produced a minimal signal response to both Gly and the four analogues. The results of the selectivity experiment indicated that the sensor displayed high selectivity for Gly.

### 3.7. Reproducibility, Repeatability, and Stability

The reproducibility of the fabricated sensor was evaluated using six distinct MIP sensor electrodes, with three independent measurements conducted. Six electrodes were fabricated under identical conditions and utilized for the detection of Gly under the same parameters. Figure 5i demonstrates that the six electrodes exhibit strong reproducibility, with an RSD of 3.35%.

Furthermore, to assess the repeatability of the sensor, a MIP electrode was employed for continuous adsorption and elution, demonstrating the capability to be repeated up to six times with an RSD of 5.0%, thereby confirming the sensor’s repeatability.

The stability of the sensor was assessed by storing the prepared electrode at 4 °C for 10 days, with evaluations conducted every two days. The electrode’s current loss remained below 10% during this period, indicating that the modified electrode exhibits good stability.

### 3.8. Real Sample Analysis

In order to verify the possibility of the sensor in practical application, the standard addition method was adopted to verify the application performance of the actual sample. Actual samples of Radix angelicae sinensis, Manyinflorescenced Sweetvetch Root, and Licorice root were utilized, with different amounts of Gly added accordingly, followed by testing with the constructed sensor, as illustrated in Table 2. The results indicate that the recovery rate of the sensor is between 94.47 and 112.23%, and the RSD is between 1.37 and 3.01%. It shows that the sensor has good application potential in practical samples.

## 4. Conclusions

This study presents a high-performance molecularly imprinted electrochemical sensor for glyphosate detection, utilizing an integrated strategy that combines rational design with sustainable materials. The sensor architecture features a biomass-derived porous carbon substrate with optimized electronic properties, complemented by a DFT-guided molecular recognition interface and an environmentally friendly elution protocol using DES. DES facilitates template removal by penetrating and mildly swelling the polymer matrix, while their potent hydrogen-bond donor and acceptor competitively disrupt template–monomer interactions and solvate the template into the eluent phase. Compared to conventional organic solvents or harsh acidic or basic eluents, DESs are particularly effective for polar, multifunctional templates, operating under milder conditions that better preserve imprint integrity. The resulting sensor achieves a remarkable detection limit (8.8 × 10^−10^ M) across a broad linear range (1 × 10^−9^–1 × 10^−6^ M), coupled with high reproducibility, repeatability, and storage stability. More importantly, its successful application in complex TCM samples, yielding accurate recoveries (94.47–112.23%), validates its practical utility. Given this, the proposed sensor shows great potential for Gly determination in TCM samples, particularly in complicated backgrounds.

## Figures and Tables

**Table 1:** Performance comparison of MIP-based electrochemical sensors for glyphosate.

Electrodes	Modified	Technique	Linear Range (M)	LOD (M)	Sensor Application	Year/Ref.
GCE	MIP/Y-BDC-NH_2_@GO ^1^	DPV	1 × 10^−9^–1.65 × 10^−5^	4.2 × 10^−10^	Tap water, agricultural water, and agricultural soil samples	2026 [7]
SPE	MIPPy ^2^/Au	CV	3 × 10^−11^–3 × 10^−10^	9.5 × 10^−12^	Water	2024 [43]
Pt-SPE	MIP nanoparticles	DPV	2.5 × 10^−11^–5.0 × 10^−10^	1.8 × 10^−11^	River water	2023 [16]
GCE	MIP/MWCNTs-Au	CV	1.0 × 10^−8^–2.4 × 10^−6^	1.4 × 10^−9^	Fruits	2023 [38]
GSPE ^3^	MSN-PtNPs@MIP ^4^	DPV	2.5 × 10^−8^–5.0 × 10^−4^	4.0 × 10^−9^	Reservoir water, pond water, waste water	2022 [44]
NRAs ^5^	Ni/MIP-NiAl-LDH	DPV	1 × 10^−8^–1 × 10^−6^	3.1 × 10^−9^	Pond water	2022 [45]
GCE	MIP@GO	DPV	0–1.8 × 10^−3^	1.1 × 10^−5^	Corn	2022 [46]
Au	MIPPy	DPV	2.9 × 10^−8^–4.7 × 10^−6^	1.6 × 10^−9^	Cucumber and tap water	2017 [47]
ITO ^6^	AuNp/MIP	PV	2.4 × 10^−9^–7.1 × 10^−9^	5.4 × 10^−10^	Corn	2017 [48]
GCE	MIP/PBC	DPV	1.0 × 10^−9^–1.0 × 10^−6^	8.8 × 10^−10^	TCM samples	This study

^1^ graphite oxide, ^2^ molecularly imprinted polypyrrole nanotubes, ^3^ graphite screen printed electrode, ^4^ molecularly imprinted polymer coated on mesoporous silica-platinum,^5^ Nanorod Arrays, ^6^ Indium tin oxide glass.

**Table 2:** Recoveries of the constructed MIP sensor in TCM samples.

Real Sample	Added (M)	Found (M)	Recovery (%)	RSD(%, *n* = 3)
Radix angelicae sinensis	5.00 × 10^−7^	4.86 × 10^−7^	97.17%	1.37
	5.00 × 10^−8^	5.23 × 10^−8^	104.60%	2.20
	5.00 × 10^−9^	5.27 × 10^−9^	105.37%	2.52
Manyinflorescenced Sweetvetch Root	5.00 × 10^−7^	4.72 × 10^−7^	94.47%	2.03
	5.00 × 10^−8^	5.61 × 10^−8^	112.23%	2.52
	5.00 × 10^−9^	5.31 × 10^−9^	106.27%	1.69
Licorice root	5.00 × 10^−7^	5.18 × 10^−7^	103.67%	0.69
	5.00 × 10^−8^	4.76 × 10^−8^	95.17%	2.65
	5.00 × 10^−9^	5.43 × 10^−9^	108.63%	3.01

## Data Availability

Data is contained within the article and Supplementary Materials.

## Supplementary Materials

The following supporting information can be downloaded at https://www.mdpi.com/article/10.3390/polym18010021/s1. Figure S1: SEM images of PBC and corresponding elemental mapping images; Table S1: Elemental composition of the PBC obtained from XPS results; Figure S2: C 1s XPS spectra of PBC; Figure S3: The infrared spectrum of PBC reveals: 3438.5 cm^−1^, indicative of O-H stretching vibrations, signifying hydroxyl groups; 1639.3 cm^−1^, associated with C=C backbone vibrations, denoting a sp^2^ hybridized structure; 1074.6 cm^−1^, corresponding to C-O stretching vibrations, indicating the existence of oxygen-containing functional groups; and 785.6 cm^−1^, related to C-H out-of-plane bending vibrations of the aromatic ring, confirming the aromatic ring structure within the carbon material; Figure S4: Pore size distribution of PBC indicates the presence of mesoporous structure.
