# Supplementary material for: Rational Design of a Molecularly Imprinted Sensor on a Biomass Carbon Platform for Glyphosate Monitoring in Traditional Chinese Medicines
Source: Polymers (Basel). 2025 Dec 22;18(1):21. doi: 10.3390/polym18010021 (PMC12787997; doi:10.3390/polym18010021)
Supplement: Supplementary file 1 [file polymers-18-00021-s001.zip › polymers-4032314-supplementary.pdf]

Supplementary Information

Rational Design of a Molecularly Imprinted Sensor on a Biomass Carbon Platform for Glyphosate Monitoring in Traditional Chinese Medicines

Xin Wang [1†], Delai Zhou [1†], Xuxia Liu [1†], Guodi Lu[1], Jia Hou [1], Jian Xu [2] and Fude Yang [1,*]

[1] School of Pharmacy, Gansu University of Traditional Chinese Medicine, Lanzhou 730000, China;
[2] Research Center for Natural Medicine and Chemical Metrology, Lanzhou Institute of Chemical Physics, Chinese Academy of Sciences, Lanzhou 730000, China; xujian1980@licp.cas.cn(J.X.);
* Correspondence: gszyyfd@163.com; Tel.: +86-0931-5162435;
† These authors contributed equally to this work.

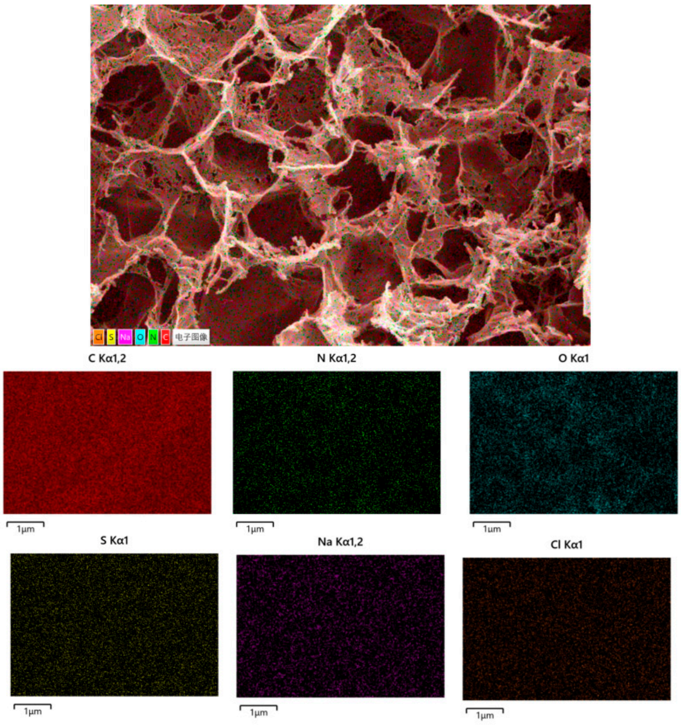

Figure S1. SEM images of PBC and corresponding elemental mapping images.

Table S1 Elemental composition of the PBC obtained from XPS results.

Sample	C (at,%)	N (at,%)	O (at,%)	Na (at,%)	S (at,%)	Cl (at,%)
PBC	89.26	3.42	5.82	0.04	0.71	0.75

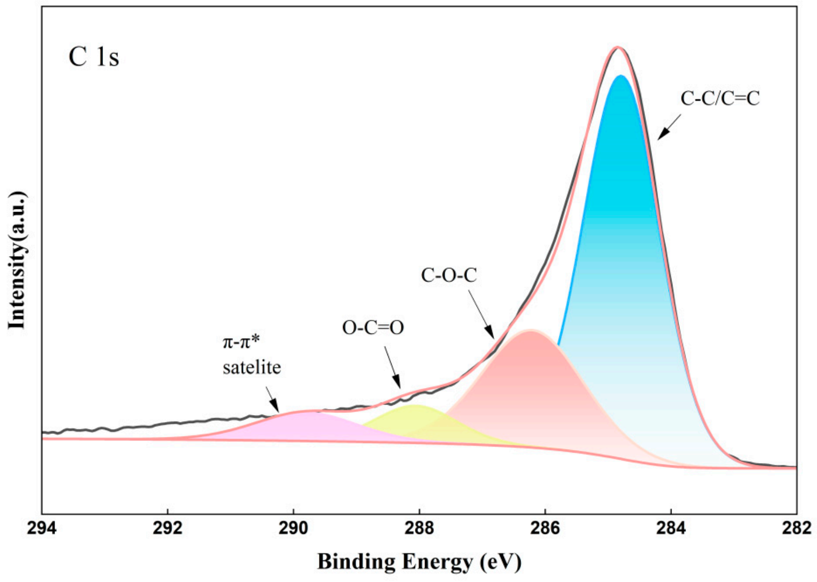

Figure S2. C 1s XPS spectra of PBC.

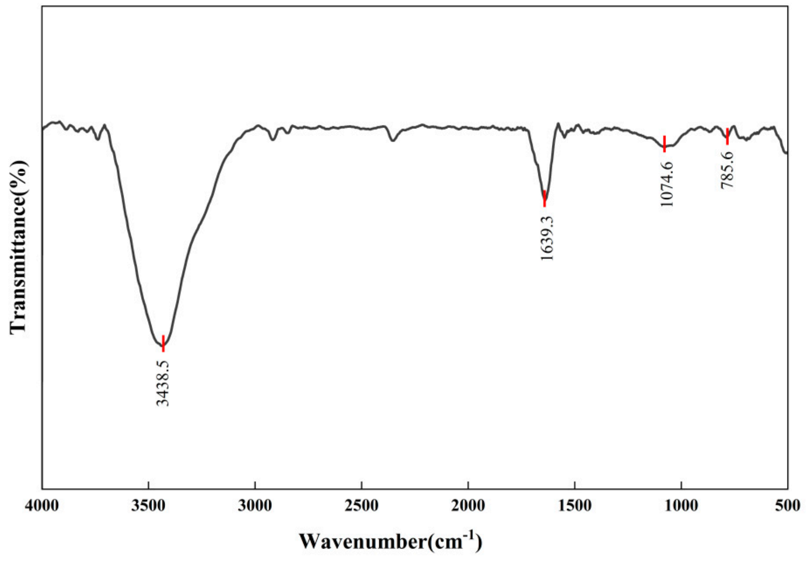

Figure S3. The infrared spectrum of PBC reveals: 3438.5 cm^{-1}, indicative of O-H stretching vibrations, signifying hydroxyl groups; 1639.3 cm^{-1}, associated with C=C backbone vibrations, denoting a sp^2 hybridized structure; 1074.6 cm^{-1}, corresponding to C-O stretching vibrations, indicating the existence of oxygen-containing functional groups; and 785.6 cm^{-1}, related to C-H out-of-plane bending vibrations of the aromatic ring, confirming the aromatic ring structure within the carbon material.

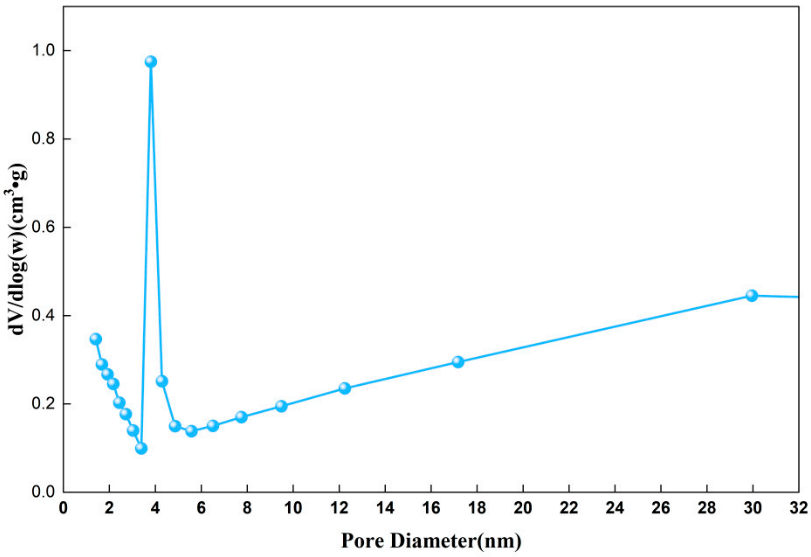

Figure S4 Pore size distribution of PBC indicates the presence of a mesoporous structure.
